# Early real-time estimation of the basic reproduction number of emerging or reemerging infectious diseases in a community with heterogeneous contact pattern: Using data from Hong Kong 2009 H1N1 Pandemic Influenza as an illustrative example

**DOI:** 10.1371/journal.pone.0137959

**Published:** 2015-09-15

**Authors:** Kin On Kwok, Bahman Davoudi, Steven Riley, Babak Pourbohloul

**Affiliations:** 1 School of Public Health, Li Ka Shing Faculty of Medicine, The University of Hong Kong, Hong Kong Special Administrative Region, Hong Kong, People’s Republic of China; 2 Division of Mathematical Modeling, British Columbia Centre for Disease Control, Vancouver, British Columbia, Canada; 3 MRC Centre for Outbreak Analysis and Modelling, Department for Infectious Disease Epidemiology, Imperial College London, United Kingdom; 4 School of Population & Public Health, Faculty of Medicine, University of British Columbia, Vancouver, British Columbia, Canada; The University of Melbourne, AUSTRALIA

## Abstract

Emerging and re-emerging infections such as SARS (2003) and pandemic H1N1 (2009) have caused concern for public health researchers and policy makers due to the increased burden of these diseases on health care systems. This concern has prompted the use of mathematical models to evaluate strategies to control disease spread, making these models invaluable tools to identify optimal intervention strategies. A particularly important quantity in infectious disease epidemiology is the basic reproduction number, R_0._ Estimation of this quantity is crucial for effective control responses in the early phase of an epidemic. In our previous study, an approach for estimating the basic reproduction number in real time was developed. This approach uses case notification data and the structure of potential transmission contacts to accurately estimate R_0_ from the limited amount of information available at the early stage of an outbreak. Based on this approach, we extend the existing methodology; the most recent method features intra- and inter-age groups contact heterogeneity. Given the number of newly reported cases at the early stage of the outbreak, with parsimony assumptions on removal distribution and infectivity profile of the diseases, experiments to estimate real time R_0_ under different levels of intra- and inter-group contact heterogeneity using two age groups are presented. We show that the new method converges more quickly to the actual value of R_0_ than the previous one, in particular when there is high-level intra-group and inter-group contact heterogeneity. With the age specific contact patterns, number of newly reported cases, removal distribution, and information about the natural history of the 2009 pandemic influenza in Hong Kong, we also use the extended model to estimate R_0_ and age-specific R_0_.

## Introduction

The emergence and global spread of a novel H1N1 influenza A virus (pH1N1) in early 2009 presented a number of new challenges to policy makers and epidemiologists [1]. In the early phases of the epidemic, with limited information on the number of infections, policy makers tried to mitigate disease spread as best they could. Mathematical modeling of infectious disease has made increasing contributions to public health decisions [2] [3] [4] with the aim of estimating the basic reproduction number, R_0_ [5]. R_0_ is defined as the number of secondary cases generated by one infectious individual during his/her entire infection period in a totally susceptible population [6]. When R_0_ is greater than one, there is a likelihood that the infection will to continue to spread; however if R_0_ is less than one, then the infection will eventually die out. For this reason, estimating the basic reproduction number is paramount for assessing the effectiveness of different intervention strategies.

Most classical mathematical models of infectious disease transmission, also known as compartmental models, assume homogeneous mixing among individuals. In other words, each individual has an equal chance of infecting others in a population. However, the estimates of R_0_ from models with this parsimonious assumption may differ greatly from models featuring heterogeneous contacts patterns, resulting in different epidemiological outcomes under the same intervention policies [7]. Studies have suggested that many infectious diseases exhibit heterogeneity in transmission efficiency in certain individuals (for example “super-spreaders” in SARS (2003)), or in specific groups of individuals that appear to account for most of the transmission (such as school age children in H1N1 influenza A (2009)) [8] [9]. To ensure that this heterogeneity is modeled, contact network models are used to explicitly capture patterns of disease transmission. Contact network models can also be used to compare the intervention strategies for different infectious diseases when epidemiological data are limited, as was the case with SARS in 2003 and H1N1 pandemic influenza virus in 2009 [5] [7].

Heterogeneity in contact patterns among individuals in different age groups is an important factor in the transmission of infectious diseases. In this study, a new approach to network modeling is outlined to demonstrate how well the real time estimation of R_0_ can be obtained in the early stages of a disease outbreak, using simulated data. Our approach will specifically take into account different levels of inter- and intra-age group heterogeneity. We also validate our analytical method using simulation data. Lastly, we use the tested methodology to estimate the basic reproduction number of pH1N1 in Hong Kong.

In the following section, we first introduce the homogeneous model (model A) without age-structure and then heterogeneous model (model B) that includes age structure. We then compare the performance of these two models using the simulation data in which inter- and intra-degree can be changed. We show that the second model converges very faster to real R_0_ value when inter-degree is big. We finally use both models to estimate the R_0_ value for the last pandemic data which occurred in Hong Kong.

## Methods

### a) Overview of a Network Approach to Estimate R_0_

Contact network models characterize the contacts between individuals in a social setting that can potentially result in disease transmission. The contact patterns can vary between individuals in the population. We assume that there are *N* individuals in the population. Each individual is represented as a vertex, and an edge represents a pathway of possible infection transmission between two individuals. The number of contacts that an individual has is denoted by *k*., Also, the probability that a randomly chosen vertex has *k* contacts (*k* degrees) is denoted *p*_*k*_. Useful information can be derived from the distribution of number of contacts, known as the degree distribution. For instance, the first moment of degree distribution is the average number of nearest neighbours to a randomly selected individual *z*_1_, and the second moment is related to *z*_2_, the number of second-degree neighbours (i.e., neighbours of neighbours) of a randomly chosen individual. The excess degree *z* is defined as the number of edges emanating from an infected individual, excluding the edge that was the source of the focal infection; it is simply the ratio $\frac{z_{2}}{z_{1}}$ [10].

A continuous time approach to estimate ongoing basic reproduction number with three-state (susceptible, infected and removed) framework was previously described in [11]. Age structure was not explicitly included in this framework. The excess degree $\frac{z_{2}}{z_{1}}$ was assumed to be the same to every individual. We defined *λ* as the transmission rate of an infected individual to a susceptible individual if the individual was not infected previously. Thus, *T*(*t*) = 1 − *e* ^−*λt*^ is the probability that an individual transmits the disease to one of its neighbours by time *t* (since acquiring the infection). In the same manner, *λ*_*r*_ was defined as the removal rate of an infected individual given that the individual was not removed from the infectious state, previously.

Thus $\psi (t)=e^{-\lambda_{r}t}$ and $\phi (t)=\lambda_{r}e^{-\lambda_{r}t}$ are probability function that the time for an individual to be removed at time *t* and its corresponding probability density function respectively. The basic reproduction number can be written as the product of the expected excess degree and the expected transmissibility $R_{0}(t)=\frac{z_{2}}{z_{1}}T(t)$.

### b) Non-Age-Structured Model (Model A)

In this study, model A is simply the discretized version of the model in [11] when the approach is applied to daily count of incidence cases. A discrete-time approximation described in [5] is used throughout the derivation of the formula of R_0_. For ease of illustration of model A, we assume the discrete time step Δ*t* to be 1 day.

Given limited information about the natural history of newly emerging diseases, *λ* and *λ*_*r*_ were assumed to be constant. As the outbreak progresses, the number of susceptible *S*(*t*), infected *I*(*t*), and recovered *R*(*t*) individuals will vary simultaneously.

However, we cannot obtain these quantities directly, and the only available information during the epidemic is the number of newly reported cases at time t, denoted as *J*(*t*).

Using *J*(*t*). and *ψ*(*t*), the following equations can be obtained (discrete-value version of [11])

$$R(t)=\sum_{i=0}^{t}\left\{1-\psi (i)\}J(t-i\right)$$

$$I(t)=\sum_{i=0}^{t}\psi (i)J(t-i)$$

The expected number of infectious and removed individuals who were infected by individuals already removed at time t, *I* ^*r*^(*t*), and *R* ^*r*^(*t*) respectively, can be estimated by *ϕ*(*t*), *J*(*t*) and *ψ*(*t*) as follows [11]:

$$I^{r}(t)+R^{r}(t)=\sum_{k=0}^{t}J(k)\left[1-\frac{\sum_{i=0}^{k}J(k-i)\psi (t+i-k)\phi (t)}{\sum_{i=0}^{k}J(k-i)\psi (i)\phi (i)}\right]$$ (1)

At time *t*, the expected transmissibility of infectious individuals before they are removed can be calculated as

$$T^{r}(t)=\frac{\sum_{i=0}^{t}T(i)\phi (i)C(t-i)}{\sum_{i=0}^{t}\phi (i)C(t-i)}$$ (2)

where $C(t)=\sum_{i=0}^{t}J(i)$ is the cumulative number of new infections at time *t*.

There is an important relationship between *T* ^*r*^(*t*), $\frac{z_{2}}{z_{1}}$, *I* ^*r*^(*t*), *R* ^*r*^(*t*), and *R*(*t*) as in [11]:

$$T^{r}\left(t\right)=\frac{I^{r}\left(t\right)+R^{r}\left(t\right)}{R\left(t\right)\frac{z_{2}}{z_{1}}}$$ (3)

By Eq (3), *λ* can be estimated and we have *T*(*t*). Given that there is available information on the number of new infections up to time *t*, the basic reproduction number can be obtained at time *t* by the following formula

$$R_{0}(t)=\frac{z_{2}}{z_{1}}\sum_{i=0}^{t}\phi (i)T(i)$$ (4)

### c) Age-Structured Model (Model B)

Model B is a modified version of model A, where age specific contact patterns are included. We defined *k* age groups with *n* individuals in the population. The excess degree of *i*th age group *z*(*i*) for *i* = 1, 2, …, *k* is calculated based on the degree distribution of *n*(*i*) individuals in *i*th age group. During the epidemic, we have information on the total number of new infections stratified by *k* age groups at time *t*. *J* ^*B*^(*i*, *t*) is defined as the total number of new infections in *i*th age group at time *t*. Thus, the excess degree of an infected population will vary across time and is defined as follows:

$${\frac{z_{2}}{z_{1}}}_{B}(t)=\frac{\sum_{i=1}^{k}z(i)J^{B}(i,t)}{J(t)}$$

Eq (3) then becomes

$$T^{r}(t)=\frac{I^{r}(t)+R^{r}(t)}{\sum_{i=0}^{t}\left\{1-\psi (i)\}J(t-i\right){\frac{z_{2}}{z_{1}}}_{B}(t-i)}$$ (5)

Using Eq (5) and $\frac{z_{2}}{z_{1}}=\frac{\sum_{i=1}^{k}n(i)z(i)}{n}$ we estimate *λ*. The basic reproduction number from model B can then be obtained numerically by using Eq (4) [12].

### d) Simulation of Incidence Data with Known R_0_ and Overall Degree Distribution

The time series data of the daily incidence of influenza is first simulated based on the binomial random network in [5] [11]. An overall degree distribution, a known R_0_ or *λ*_*r*_ and the natural history of influenza are inputs of the network. We assume that the contact patterns of individuals would be similar to those seen in a typical day in the early phase of the epidemic.

To evaluate which model can rapidly provide a better estimate of R_0_ in the early phase of an epidemic of influenza, the same overall degree distribution which is the same as simulated data and the number of daily reported cases are inputs of both model A (without age structure) [5] and the proposed model B (with age structure). Ongoing estimate of R_0_ from each model will be provided. We also apply model B to the real data as an illustrative example with 2 age groups: school aged children (<20 years old) as age group 1 and adults (20 years old or above) as age group 2 to estimate R_0_ for the 2009 outbreak of H1N1pdm in Hong Kong [S1 Data]. From the start of May in 2009, patients who were admitted to public hospitals with acute respiratory illness were tested by (RT)-PCR. Information from each individual tested was entered into an information system (E-flu) managed by the Hong Kong Hospital Authority–a statutory body responsible for managing Hong Kong's public hospitals and their services to the community. The E-flu database generates incidence data on children and adults by the date of symptoms onset. This database was gathered as a part of a public health response and therefore not subject to any ethics approval. Permission was granted to analyze these data anonymously for the purpose of this study.

## Results

We used a time step in our model of 0.02 days. The two models were tested under different levels of intra- and inter-age group contact heterogeneity in different sizes of epidemics. For ease of illustration, only two age groups in the community are considered (adults and children), although the above equations could easily be generalized to situations where information about more refined age groups is available. With six different known values of R_0_ and similar value for *λ*_*r*_, we used computer simulations to generate incidence data for these two age groups under three levels of inter-group and within group heterogeneity in contact patterns. In these simulations we construct contact patterns such that individuals in each age group have uniform degree distributions with a given width.

**Fig 1:**
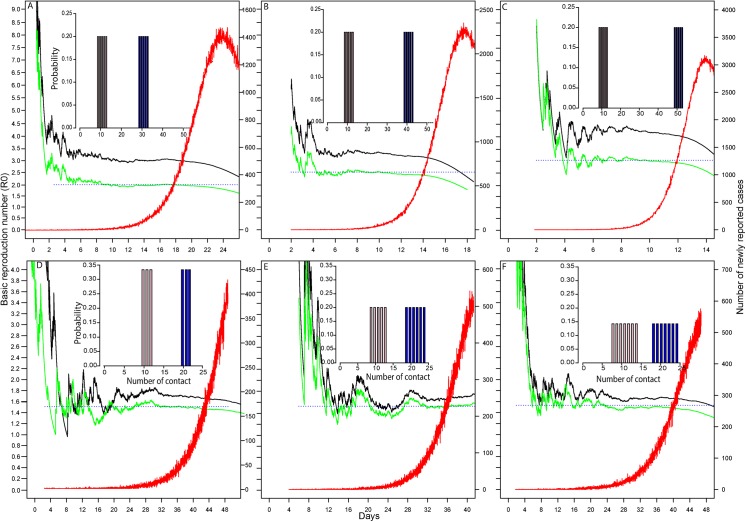
Real-time estimation of R_0_ using model A (black) and model B (green). Both models are run under different hypothetical degree distributions and two known values of R_0_ for the two age groups (shown in the sub-diagrams in the initial phase of the epidemic). The true values of R_0_ (Fig 1A-1F) are 2.05, 2.58, 3.12 1.51, 1.52 and 1.53 respectively. The red line shows the epidemic curve and the dotted blue line is the true value of R_0_. The corresponding values of *z*_*2*_ */z*_*1*_ are (A) 19.1, (B) 24.1, (C) 29.1, (D) 14.0, (E)14.15, (F)14.3.

Similar to assumptions in [11], we defined the value of *λ*_*r*_ as 0.25, an arbitrary value for generating synthetic incidence data. Six known values of R_0_ (Fig 1A–1F) are 2.05, 2.58, 3.12 1.51, 1.52 and 1.53, respectively. In Fig 1A, we show that model B with age structure yields a better estimate of R_0_ and converges at the true value (R_0_ = 2.05) more quickly in the early phase of the epidemic (~day 9 to 15) over non-age-structured model A (day 18 to day 21). Between day 9 and day 15, the estimated R_0_ values fluctuate ~ 0.4% around the true value. Between day 0 and day 15, less than 100 cases are required to estimate R_0_.

In densely populated area like Hong Kong, the between group and within-group variation in number of contacts is very high. To characterize this feature, we hypothesized 2 scenarios for 2 given values of R_0_ as seen in Fig 1A–1C and Fig 1D–1F. Across Fig 1A–1C, we keep the degree distribution of individuals in the first age group unchanged (red bar chart in the sub-diagram) and shift the degree distribution of individuals in the second age group (blue bar chart in the sub-diagram) to the right, thereby increasing the inter-group heterogeneity of contacts. Model B shows a quick convergence to the true value of R_0_, while model A cannot accurately estimate R_0_, especially when inter-group heterogeneity in the contact network is high. This suggests that model B provides a better real-time estimate over model A whenever there is any level of inter-group contact heterogeneity.

We also test which model gives a more accurate estimate of R_0_ with larger intra-group contact heterogeneity (a wider distribution in each age group). We run the simulations on each age group using three datasets where the contacts are uniformly distributed with a width of 3, 5, and 7 degrees respectively (Fig 1D–1F). For incidence data with 3 degrees on each group, model B provides more accurate estimates of R_0_ between day 30 and day 45 than model A (as in Fig 1D). Under these conditions, about 50 to 100 cases are required for model B to converge on the true value of R_0_.

For incidence data with 5 degrees and 7 degrees on each group, model B also gives an accurate estimate of R_0_ as represented in Fig 1E and Fig 1F. In Fig 1F, the distance between degree distributions of the two age groups is shortened compared with Fig 1D. In other words, the inter-group contact heterogeneity in the two age groups decreases across Fig 1D–1F. This explains why the differences between the two estimates from both model A and model B are not significant in Fig 1F. It also reveals that model A is a good choice if contacts are more uniformly distributed across different age groups.

**Fig 2:**
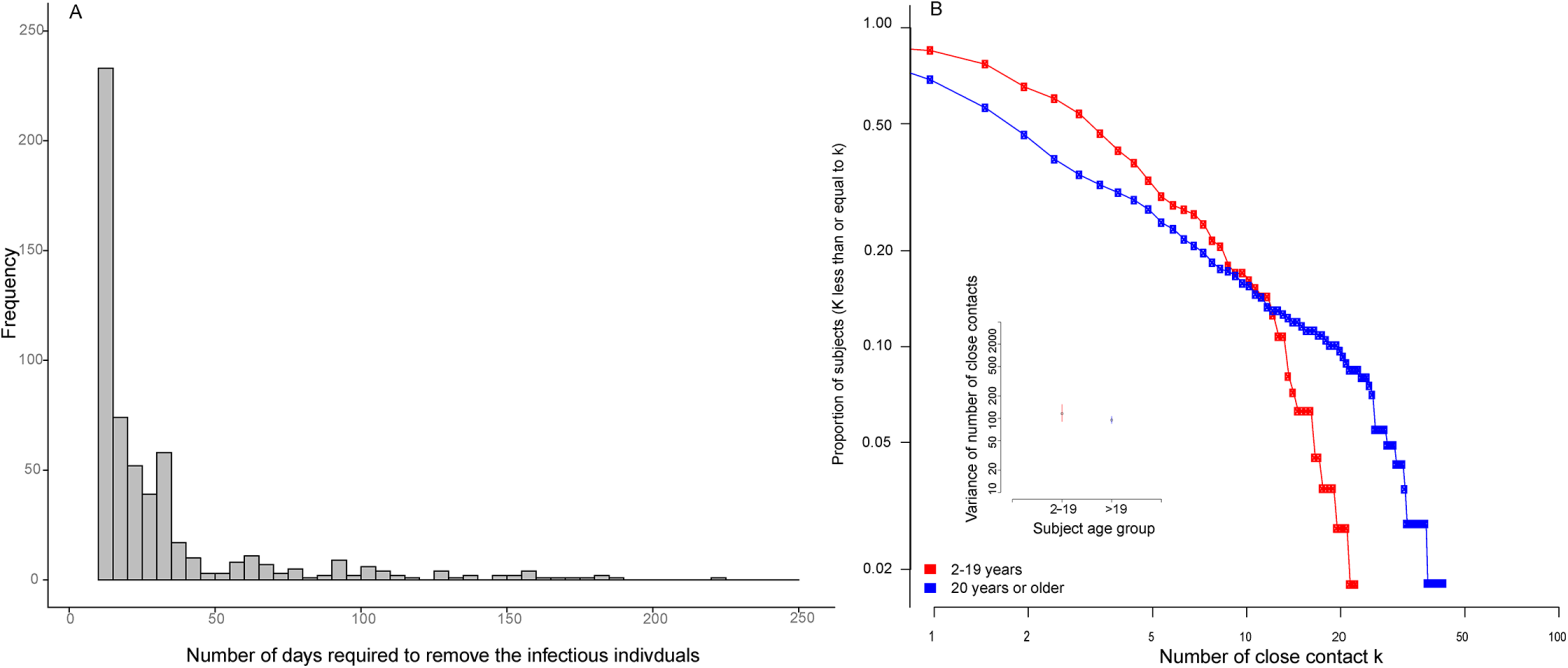
Removal distribution and inverse cumulative function of number of close contacts. **(A)** Frequency distribution of the number of days required to remove infectious individuals after the date of symptoms onset, separated by age group (2–19 or 20 and above). (B) Inverse cumulative function of the number of close contacts, point estimates, and 95% confidence intervals for the variance in the number of close contacts in individuals aged 2–19 (children) and 20 or above (adults).

We also applied model B to the hospital incidence data extracted from the E-flu system and the overall degree distribution from the contact survey to estimate R_0_ for the 2009 outbreak of H1N1pdm in Hong Kong. In total, there were 28,338 laboratory-confirmed cases during the period between 30^th^ April 2009 and 7^th^ February 2010. The removal distribution can also be derived using the 24,873 laboratory-confirmed cases with complete information on symptomatic notification date and symptoms onset date (Fig 2A). The median number of days to be removed is 1 day. Using parameter estimates determined from the natural history of pandemic influenza in 2009 [13] [14] [15] [16] as shown in Table 1, we re-defined our transmissibility function as follows,

$$T(t)=\left\{ \begin{array}{l} 0\\ 1-{\left(1-\lambda \right)}^{t-\Delta l+1}\\ 1-{\left(1-\lambda \right)}^{\Delta i+\Delta s} \end{array}\right.\begin{array}{c} t<\Delta l\\ \Delta l\leq t<\Delta l+\Delta i+\Delta s\\ t\geq \Delta l+\Delta i+\Delta s \end{array}$$

where Δ*l*, Δ*s* and Δ*i* represent the average duration of the latent phase of infection, average duration of the asymptomatic phase of infection, and the average duration of the symptomatic phase of infection respectively.

**Table 1:** Parameter values used and estimates in model A and model B.

Parameter values used in Model A and B	Value	Notes
**Average duration of the latent phase of infection (Δ*l*) (days).**	3	Integer value within the 95% CI of the estimate (2.28, 3.12) from analysis of the 2009 influenza A (H1N1) pandemic in the province of Ontario, Canada [13].
**Average duration of asymptomatic phase of infection (Δ*s*) (days).**	1	[14] [15]
**Average duration of the symptomatic phase of infection (Δ*i*).**	3	Integer value within the 95% CI of the estimate (2.06, 4.69) from analysis of the 2009 influenza A (H1N1) pandemic in the province of Ontario, Canada [13].
**Parameter estimates in Model A**		
**Overall excess degree in Hong Kong**	23.0	This value is derived from the degree distribution of 770 (*n*) subjects who provided either an exact or a range number of close contacts in the interview-led questionnaire survey in the Hong Kong population-based serological study in 2009 [17], [16].
**Basic reproduction number (R_0_)**	1.21	This value is extracted from the flat region in Fig 3 from Day 82 to Day 102, after the first laboratory confirmed case on 30^th^ April, 2009 (blue line in Fig 3).
**Parameter estimates in Model B**		
**Excess degree of children (less than 20 years old) in Hong Kong.(*z*(1))**	22.4	This value is derived from the degree distribution of 112 (*n(1)*) children who provided either an exact number of close contacts (n = 99) or a range number of close contacts (n = 13) on a pre-assigned random day in the interview-led questionnaire survey in the 2009 Hong Kong population-based serological study [17], [16].
**Excess degree of adults (20 years old or above) in Hong Kong. (*z*(2))**	18.9	This value is derived from the degree distribution of 658 (*n(2)*) adults who provided either an exact number of close contacts (n = 611) or a range number of close contacts (n = 47) for a pre-assigned random day in the interview-led questionnaire survey in the 2009 Hong Kong population-based serological study [17], [16].
**Overall excess degree in Hong Kong**	19.6	This value is derived from the weighted average of z(1) and z(2) and the proportion of the two age groups in the Hong Kong population in 2009 as per census and statistics department information.
**Basic reproduction number (R_0_): i) Overall; ii) Children; iii) Adults**	1.12; 1.28; 1.08	This value is extracted from the flat region in Fig 3 from Day 82 to Day 102, after the first laboratory confirmed case on 30^th^ April, 2009.

We derived the excess degree and degree distribution for each age group from an analysis of the number of close contacts, *k*, experienced by 770 participants aged 3 to 102 years taken from the interview-led social contact questionnaire in [17] [18]. The subjects in this study were required to record detailed contact histories for one pre-assigned day, providing information on how many contacts they had made, whom they had met, and how long they had met with each contact. Out of 770 subjects, 710 subjects recorded how many close contacts they had made. The rest of the 60 subjects provided a range in the number of close contacts they had experienced, and we defined their number of close contacts as the mid-point of this range. The mean number of close contacts for school-aged children and adults was 9.94 and 4.69 respectively. The degree distribution for individuals aged 2–19 is different from those aged 20 and above as shown in Fig 2B. In the sub-diagram of Fig 2B, the variance in the number of contacts of children and adults is 115.7 [90.4, 153.4] and 94.7 [85.3, 105.9] respectively. This shows that there is higher variation in the number of contacts in children than in adults.

**Fig 3:**
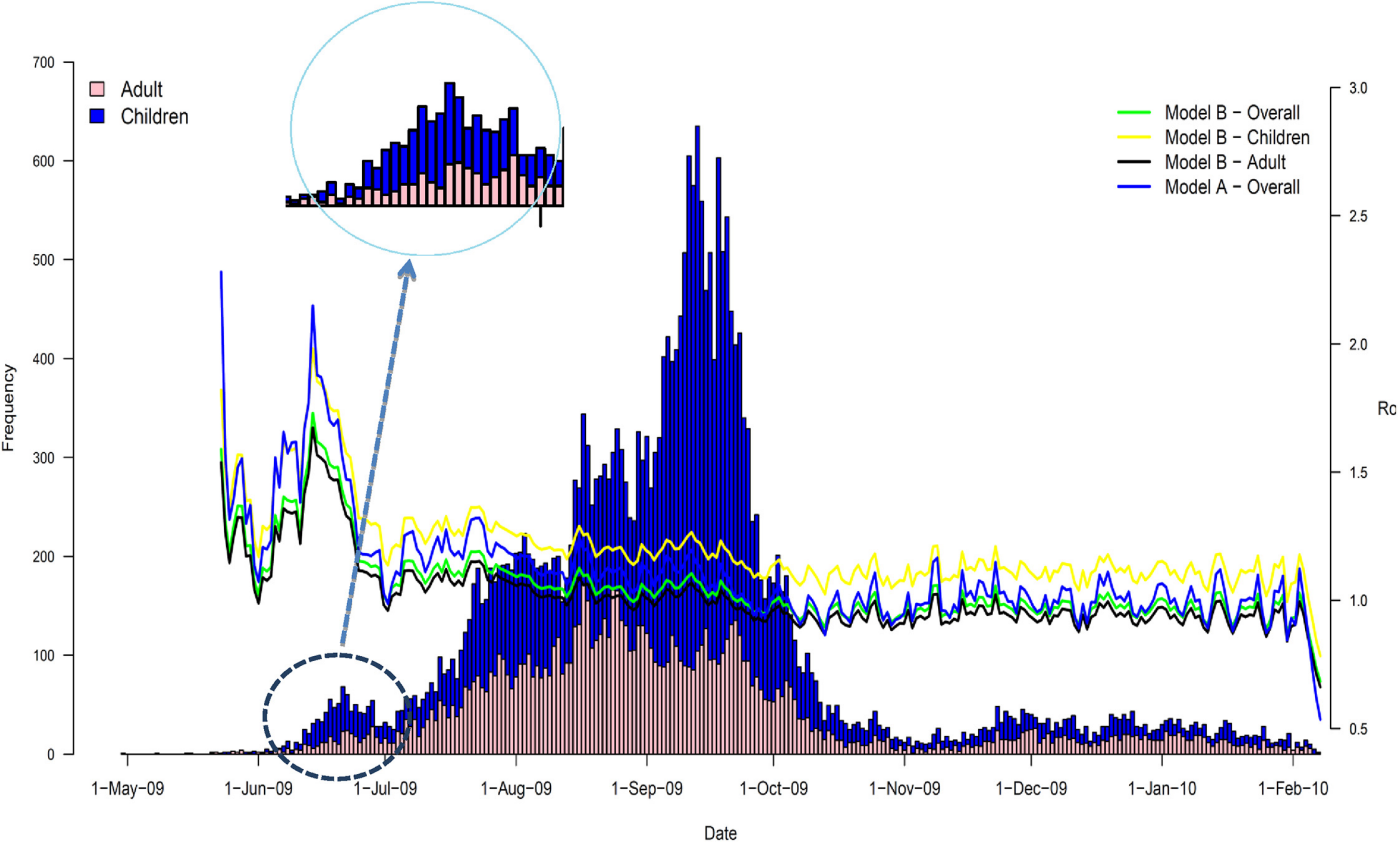
Incidence of laboratory confirmed H1N1pdm cases during the 2009 pandemic influenza and real time estimation of R_0_. Stacked bar chart shows the incidence of laboratory confirmed H1N1pdm cases in individuals aged 2–19 years and aged 20 or above. The line chart shows the real time estimation of R_0_ using model B.

Fig 3 reveals that while the daily incidence of laboratory-confirmed cases of pH1N1 among adults in Hong Kong increased monotonically from April until the main peak in September 2009, the incidence in children presented an anomaly. The incidence data for children has a minor peak at the early stage of the epidemic between June and July. In real time, at the time when the minor peak formed, and without prior knowledge of how the epidemic would unfold afterwards, this anomaly might have been interpreted as a stand-alone peak, with an inclining segment between June 1 and 21 and a declining segment between June 21 and July 1 as shown in the inset figure in Fig 3. Interestingly, using the inclining segment with model B, the estimate of R_0_ converges very quickly to 1.2 in early June (black line in Fig 3). Since the declining segment destabilizes the early-stage R_0_ estimator, the estimate after the minor peak fluctuates for a while until the epidemic curve begins to increase monotonically in July, when the estimator converges again to 1.12 between day 82 and day 102. The estimate of R_0_ under model A converges to 1.21 in late August (Day 115), which is slightly higher than the estimate from model B. Estimates under both model A and B are similar to the estimate of the effective reproduction number between 1.1 and 1.2 that was proposed in summer of 2009 by [18]. The number of cases required for model B to converge to estimate R_0_ of 1.2 and 1.12 up to Day 30 and 82 are 66 and ~2200, respectively, which are about 5 to 10% of the total number of laboratory confirmed cases in the whole epidemic. Using the excess degree of children and adults, the age specific R_0_ can be estimated for both children and adults. We showed that R_0_ for children is higher than that for adults (1.28 versus 1.08), which is consistent with other studies (see [19]). This implies that school-aged children likely contributed more infections than adults.

## Discussion

In this paper, we examined the rate of convergence when estimating the basic reproduction number, R_0_, from case notification data during the early stage of an infectious disease outbreak. By explicitly taking in account the age structure of a population, this study improves on a network-based formalism that was introduced previously for a non-age-stratified population in [11]. Our proposed age-structured model (model B) provides a reliable estimate of R_0_ in the early stage of an epidemic, even in the presence of noise or dominant stochastic fluctuations and potentially biased numbers of newly reported cases during the early stochastic regime. Using the simulated data, our analytical justification shows that network structures featuring inter-age group contact heterogeneity helps us provide an ongoing estimate of R_0_ before the epidemic reaches the exponential growth as in Fig 1. Even for the real data, our proposed model B can still provide estimates of R_0_ in the early stage of epidemics as of Day 30. Although the first peak deviates from the estimate of R_0_, it quickly converges again on the final estimate two and half months before the second peak in mid-September. The ongoing estimate of R_0_ provides important information for policy makers to take immediate action to control further disease spread.

One of the important inputs to model B is the age-specific contact information. Thus, conducting a large-scale population-based survey on contact behaviour is suggested in every city. For densely populated cities like Hong Kong, contacts of individuals within and between different age groups may vary significantly. For this reason, a large scale population-based telephone survey in Hong Kong has recently been conducted to collect the contact histories of individuals at four different time points in a year, to examine the temporal changes of contacts, and generate unbiased estimates of contact between and among age groups [20].While model A provides a reasonable estimate for R_0_ in the general population as a whole, model B–which takes age structure into account–portrays a more refined picture of patterns of disease spread across different age groups, thus providing an age-specific estimate of R_0_. To better prepare for re-emerging and emerging infectious diseases, the use of model B and further studies on the temporal change of contacts’ behaviour in major metropolitan areas may help refine these estimates even further, and provide more accurate insight into future outbreaks of infectious diseases in the early phase.

As we have shown in Fig 1A–1C, and Fig 1D–1F, estimates from model A are sensitive to the degree of contact heterogeneity while estimates from model B are not. For simplicity, we have illustrated different scenarios of degree distribution for only two age groups to test both model A and B. However, real populations have far more complex structures with very different contact patterns. Specifically, the degree distribution of individuals in each age group is neither uniform nor non-overlapping. The analytical/numerical framework presented in this paper can be readily applied to situations where these constraints are relaxed.

Our estimate of R_0_ relies on assumptions of discrete time, constant infectivity, and removal functions. Also, there are some extrinsic factors, which may raise additional uncertainties. For example, we have not included the possibility of late reporting or underreporting of numbers of new cases in the early phases of an epidemic. The assumption that the number of reported cases accurately reflects the actual number of cases may not hold true in real situations since first, not all infected persons will seek medical attention; and second, there are strong biases for certain age groups (such as children) to seek medical attention even in a precautionary capacity. To our knowledge, this model is the first network-based modeling framework that includes heterogeneity in the estimation of R_0_. In this framework the confidence interval of R_0,_ is not constructed because the stochastic variables such as *I* ^*r*^(*t*), *R* ^*r*^(*t*), *T* ^*r*^(*t*) used from Eqs (1–5) involves inherent uncertainties in arriving the estimator of R_0_ in addition to the above extrinsic factors.

The proposed methodology in its current form, does not address an assessment of the uncertainty in the point estimate of R_0_ at any given time; to achieve a more robust policy decision-support tool, the development of accompanying methodology to determine confidence intervals should be undertaken in future work.

Despite uncertainties about non-perfect observations collected from the field during an outbreak, this approach could still make an important contribution to the estimation of the basic reproduction number in the early phases of an epidemic, assuming the proportion of underreporting is not changing substantially. Improving case notification through real-time surveillance to obtain more accurate case data, combined with the proposed modeling framework, could provide an invaluable platform to inform policy makers by estimating a more accurate R_0_ in real time, thus allowing policy makers to take immediate action to control epidemics while still in the early phases.

## Supporting Information

S1 Data Age-specific daily count of reported pH1N1 (2009) cases in Hong Kong from April 30, 2009 to February 7, 2010. (CSV)
